# Blood donation projections using hierarchical time series forecasting: the case of Zimbabwe’s national blood bank

**DOI:** 10.1186/s12889-024-18185-7

**Published:** 2024-04-01

**Authors:** Coster Chideme, Delson Chikobvu, Tendai Makoni

**Affiliations:** Department of Mathematical Statistics and Actuarial Sciences, University of the Free State, P.O. Box 339, Bloemfontein, 9300 South Africa

**Keywords:** Hierarchical forecasting, Blood donation, Top-down, Bottom-up, Optimal combinations

## Abstract

**Background:**

The discrepancy between blood supply and demand requires accurate forecasts of the blood supply at any blood bank. Accurate blood donation forecasting gives blood managers empirical evidence in blood inventory management. The study aims to model and predict blood donations in Zimbabwe using hierarchical time series. The modelling technique allows one to identify, say, a declining donor category, and in that way, the method offers feasible and targeted solutions for blood managers to work on.

**Methods:**

The monthly blood donation data covering the period 2007 to 2018, collected from the National Blood Service Zimbabwe (NBSZ) was used. The data was disaggregated by gender and blood groups types within each gender category. The model validation involved utilising actual blood donation data from 2019 and 2020. The model's performance was evaluated through the Mean Absolute Percentage Error (MAPE), uncovering expected and notable discrepancies during the Covid-19 pandemic period only.

**Results:**

Blood group O had the highest monthly yield mean of 1507.85 and 1230.03 blood units for male and female donors, respectively. The top-down forecasting proportions (TDFP) under ARIMA, with a MAPE value of 11.30, was selected as the best approach and the model was then used to forecast future blood donations. The blood donation predictions for 2019 had a MAPE value of 14.80, suggesting alignment with previous years' donations. However, starting in April 2020, the Covid-19 pandemic disrupted blood collection, leading to a significant decrease in blood donation and hence a decrease in model accuracy.

**Conclusions:**

The gradual decrease in future blood donations exhibited by the predictions calls for blood authorities in Zimbabwe to develop interventions that encourage blood donor retention and regular donations. The impact of the Covid-19 pandemic distorted the blood donation patterns such that the developed model did not capture the significant drop in blood donations during the pandemic period. Other shocks such as, a surge in global pandemics and other disasters, will inevitably affect the blood donation system. Thus, forecasting future blood collections with a high degree of accuracy requires robust mathematical models which factor in, the impact of various shocks to the system, on short notice.

## Introduction

Blood transfusion requirements are on the rise globally as a result of accidents, diseases and advanced surgeries. Zimbabwe often experiences shortages in the majority blood group O during public holiday periods. This is mainly due to the high demand for clinical blood transfusion as a result of a surge in road accidents and injuries during such periods [1]. Type O blood is the most needed blood type in transfusion centres and more than 52% of Zimbabweans are in blood group O [2]. Accurate forecasts of the number of volunteer donors and blood donations help blood service managers in managing their categories of blood inventories and plan accordingly for the education and recruitment of voluntary non-remunerated blood donors and subsequent blood collection.

In the blood supply chain, future forecasting of blood supply is a critical step to ensure the adequate availability of safe blood when clinical transfusion is required. Accurate and coherent blood donation forecasting provides blood managers with empirical evidence regarding when to order blood, educate and recruit new blood donors, the estimated quantities required of each blood group to collect and potential donor categories to target.

The blood supply chain is dynamic, and as such, some studies have expressed concern over the potential reduction in blood donations emanating from multiple factors including donor demographical variations [3]. Time series analysis can be used to understand the patterns in blood donation data and help blood managers in predicting future blood donations. This information is useful for minimising volatility in blood stocks and preventing blood stockouts. Understocking blood has detrimental effects on patient safety in the healthcare system, while overstocking results in wasteful discards of outdated blood [4–9]. Accurate and coherent forecast of blood donations are vital as part of the decision support system for blood centre authorities as they need to know the future of blood supply when given the surge in daily blood demand [10].

The blood supply chain is dependent upon a finite number of donors. This is then aggravated by the fact that blood donation is very irregular and uncertain [11]. Blood donations/demand estimates based on employee opinions, experience, and intuition rather than quantitative models are currently used to determine both the current and future blood provisions in most blood centres globally, and especially in developing countries [12]. The unavailability or non-use of quantitative models in estimating blood donations can indeed cause volatility and uncertainty in the blood supply chain. Without these models, it can be difficult to accurately predict how much blood will be available for clinical transfusions, and this can lead to shortages or excesses of blood in certain areas. The application of quantitative prediction models in a blood bank helps to reduce errors in decision-making about the quantity of blood to be supplied and demanded [13].

With increased demand for blood and blood components against a declining voluntary blood donor pool, improving the availability and safety of the blood supply, and forecasting become vital for sustaining any blood bank to meet its core mandate. Numerous techniques have been applied in time series forecasting in general, such as autoregressive integrated moving average (ARIMA), exponential smoothing (ES), fuzzy systems (FS), artificial neural networks (ANN), logistic regression, support vector machine (SVM) and hierarchical time series forecasting. Hierarchical time series forecasting allows forecasting of time series at different levels of a hierarchical structure whilst preserving the relationships and dependencies within the hierarchy. Furthermore, the forecasts at each hierarchical level are aggregated or disaggregated to give the forecasts at higher or lower levels in the hierarchy.

The correlation between data of donor specific characteristics and blood donations can result in huge datasets of times series, which can then be classified into clusters or hierarchies. The essence of hierarchical forecasting in blood donation is derived from the fact that the blood donors can be categorised into various clusters such as gender, blood group type, and donor status. Data from the NBSZ indicates that male blood donors constitute about 54% of the donor pool and the female donors accounting for the remaining 46%. Also, the donations are classified according to the ABO donor blood group system with blood group O accounting for 54%, blood group A constitutes 24%, blood group B, 18% and blood group AB, 4%.

Hierarchical time series is effective in forecasting hierarchically organised data which can be aggregated and disaggregated at different levels [14]. In the blood supply chain, the total blood donation forecast is required at the top level of the hierarchy for inventory planning, resource allocation and other blood drive logistics. It is possible to create a hierarchical structure that captures the relationships between these different categories of donors. For example, at the highest level of the hierarchy, there may be forecasts for the overall national blood supply. At the next level down, there may be forecasts for the different gender of donors, viz: male and female. At the next level down, there may be forecasts for the different blood group types, viz: A, B, AB, and O. When these different levels based on donor characteristics are not factored in, this may result in incoherent time series forecasting, less targeting of interest groups, resulting in not being able to meet blood demand of a particular type in a given area(s).

The aim of the study is to use a hierarchical time series forecasting approach to predict blood donation patterns. By using this approach, it is possible to create more accurate and detailed forecasts for blood donations, taking into account the relationships and dependencies between different categories of donors. This can help blood banks to better manage their inventory and ensure that they have enough blood for the right group, adequate number of units in an area given at the right time to meet patient needs. To the best of our knowledge, the application of hierarchical time series in the blood supply chain problems has not been investigated, especially in the context of Zimbabwe and Africa, considering the available blood supply chain forecasting literature. Hierarchical forecasting is a very instrumental statistical technique to support decision-making in most supply chains [15], hence its application in blood donation projections is vital.

## Literature review

Forecasting hierarchical time series is a relatively new to the forecasting phenomenon. Hierarchical time series forecasting has gained wider application in recent years [16]. Many phenomena in the real world, such as stock prices, weather, consumer demand, tourism demand, blood supply system, just to mention a few, can be modelled using the hierarchical time series. However, the correlation of different points of the time series makes some of the algorithms less versatile in forecasting [13]. A multivariate time-series model based on long-short-term memory (LSTM) in forecasting blood donation and demand during the Covid-19 pandemic at Tehran Blood Centre in Iran [17]. The LSTM is a recurrent neural network-based deep learning model. The study results showed that the forecasting model reduced blood shortage and wastage by 5.5% when compared to existing forecasting methods, such as the ARIMA, used the time series models in forecasting blood donation at a university medical care centre in Portugal [11]. The study developed six models, viz: ETS, Holt-Winters, autoregressive neural networks, ARIMA, double-seasonal Holt-Winters, and exponential smoothing (ES). The study concluded that trend lines of donations were better modelled by different models with different forecasting horizons. However, the ARIMA model outperformed all the other models in generating forecasts, hence the ARIMA model is part of the hierarchical forecasting approach to be adopted in this study, forecasted the supply of blood at blood centres in Taiwan using data from the Taiwan Blood Services Foundation [18]. They applied two different techniques in forecasting, viz: times series and machine learning. Under time series, they employed autoregressive (AUTOREG), ARMA, ARIMA, seasonal ARIMA (SARIMA), seasonal exponential smoothing model (ESM) and Holt-Winters. Under the machine learning algorithms, they used ANN and multiple regression. The study results showed that time series forecasting methods (seasonal ESM and ARIMA models) generated accurate predictions when compared to machine learning algorithms. Hence, this study will adopt ARIMA and ETS models, concurred that blood donation was influenced by transfusion demand [19]. The study forecasted red blood cells demand using three-time series methods, viz: ARIMA, Holt-Winters and neural-network-based method. The study results showed that a SARIMA model produced accurate forecasts over a shorter time horizon of one year. The ES outperformed the other methods over longer time horizons stated that managing blood supply and demand was difficult in most blood banks globally [20]. They highlighted the need for accurate and reliable blood supply and demand forecasting models. They conducted a study at the National Health Service Blood and Transplant in England using four different time series methods which were selected using the minimum mean squared error (MMSE) and weighted least squares error (WLSE). The methods yielded similar results.

A study concluded that there is no single statistical forecasting technique that is universally better and applicable at all times [21]. The authors conducted blood demand forecasting in Finland and the Netherlands. They applied moving averages (MA), ETS, ARIMA, autoregressive neural networks (NNAR), seasonal naïve (SNAIVE), method averaging (AVG), seasonal trend decomposition methods (STL and STLF), dynamic seasonal method (TBATS), dynamic regression (DYNREG), multilayer perceptrons (MLP) and extreme learning machine (ELM). The model performances were compared using mean absolute percentage errors (MAPEs). The results show that DYNREG performed better than the other approaches in generating forecasts emphasised the importance of accurate predictions in blood provision [22]. Their study at Shirazi blood centre in Iran applied ARIMA, ANN and hybrid approaches in forecasting different blood groups demand. Mean Square Error (MSE) and Mean Absolute Error (MAE) were used to compare and validate the fitted models. The results showed that ARIMA model outperformed the other models in the forecasting accuracy [23] forecasted the demand in the blood supply chain using platelets at Canadian Blood Services. The study used five different forecasting methods, viz: ARIMA, Prophet, lasso regression (least absolute shrinkage and selection operator), random forest and LSTM (Long Short-Term Memory) networks. The results showed that with limited data, multivariate models performed better than univariate models. However, with adequate data, ARIMA models produced similar results to multivariate methods. The current study uses both the ARIMA and the ETS under the hierarchical forecasting approach models.

## Material and methods

Secondary data used in this study corresponds to the grand total of blood collections (blood units) from the five regional blood centres in Zimbabwe based on specific donor characteristics (gender and blood group). This information is useful for developing a hierarchical structure for forecasting blood donations, as it enables the researchers to consider the relationships and dependencies between different categories of blood group types and blood donors. The data was collected from the NBSZ Laboratory Information Management System (LIMS) and annual reports which are freely available on the link https://nbsz.co.zw/, where certain blood donations information is captured in aggregate form. Monthly blood donation data covering the period 2007 to 2018 was used in the forecasting, giving a total of 144 monthly observations.

**Fig. 1:**
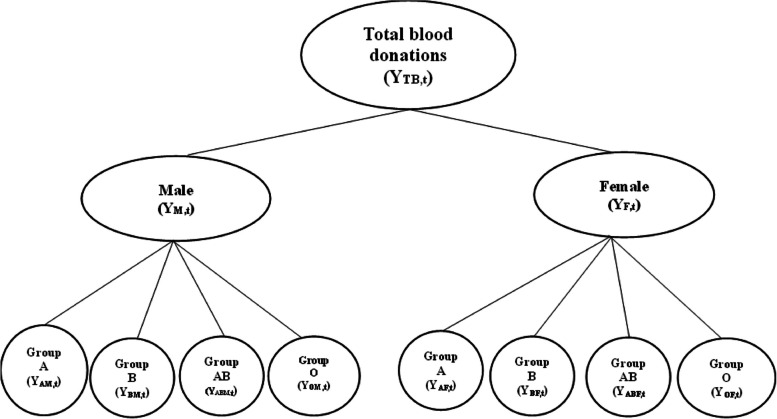
Blood donations hierarchical structure based on donor gender and blood group type

Using the approach [14], a tree diagram of blood donations comprising a two-level hierarchical structure is presented in Fig. 1. The tree diagram is constructed based on the disaggregated blood data that was categorised according to two variables: gender (Male and Female) and blood group type (A, B, AB and O). Level 0 represents the total blood donations in Zimbabwe. Level 1 denotes the first disaggregation by gender (Male (M) and Female (F)). Level 2 denotes further disaggregation by blood groups according to the ABO blood group system (A, B, AB and O).

The R-package HTS is used to generate the forecasts using the bottom-up, top-down and the optimal combination methods. The EST and ARIMA methods are used to generate the forecasts.

## Hierarchical forecasting techniques

(Fig. 1. Blood donations hierarchical structure based on donor gender and blood group type).

According to Fig. 1, level 0 gives completely aggregated blood donations (Total blood donations) denoted by ${Y}_{TB,t}$ where $t=1, 2, 3, . . . , 144$ and are obtained by adding all the series at level 1 or level 2. Level 1 represents data disaggregated according to gender (male and female). Level 1 and level 2 series can be denoted by ${Y}_{i,t}$, where $i$ denotes the node in the hierarchical tree diagram. The data consists of 144 monthly observations (t = 1, 2, …, 144). Forecasts for each level were estimated using the bottom-up, top-down and optimal combination approaches. The approach with a lower accuracy measure estimated by MAPE was used to generate forecasts for the blood centre.

Let ${{\varvec{Y}}}_{t}$ and ${{\varvec{S}}}_{11X8}$ be the vector of the blood data and a summing matrix storing the hierarchical structure shown in Fig. 1 respectively.

1 $${{\varvec{Y}}}_{t}=\left[{Y}_{TB,t}, {Y}_{M,t}, {Y}_{F,t},{Y}_{AM,t}, {Y}_{BM,t}, {Y}_{ABM,t} {Y}_{OM,t}, {Y}_{AF,t}, {Y}_{BF,t}, {Y}_{ABF,t}, {Y}_{OF,t}\right]{\prime}$$

2 $${\varvec{S}}= \left(\begin{array}{c}1 1 1 1 1 1 1 1\\ 1 1 1 1 0 0 0 0\\ 0 0 0 0 1 1 1 1\\ 1 0 0 0 0 0 0 0\\ 0 1 0 0 0 0 0 0\\ 0 0 1 0 0 0 0 0\\ 0 0 0 1 0 0 0 0\\ 0 0 0 0 1 0 0 0\\ 0 0 0 0 0 1 0 0\\ 0 0 0 0 0 0 1 0\\ 0 0 0 0 0 0 0 1\end{array}\right)$$

Making use of the summing matrix (**S**), Eq. 1 can be written as

3 $${{\varvec{Y}}}_{t}={{\varvec{S}}{\varvec{Y}}}_{2,t}$$

## The bottom-up method

The bottom-up approach involves forecasting individually for each series at the lowest levels of the hierarchy and then aggregates the forecasts upwards to generate forecasts for higher levels. The method is based on forecasting the individual blood donations from the blood group type A, B, AB and O first. Total number of blood donations for each gender can be calculated by summing up the forecasted donations made by individuals of all blood groups. Then, by summing up the donations made by each gender, one can determine the total number of blood donations for the blood bank. In other words, the approach is concerned with producing individual base forecasts at the lower level of the hierarchy and combining the forecasts upwards through ${\varvec{S}}$. Thus, the approach starts by producing *h*-step-ahead forecasts for individual bottom level time series ($n = 8$):

$${\widehat{Y}}_{AM,h},$$ $${\widehat{Y}}_{BM,h,}$$ $${\widehat{Y}}_{ABM,h},$$ $${\widehat{Y}}_{OM,h},$$ $${\widehat{Y}}_{AF,h},$$ $${\widehat{Y}}_{BF,h,}$$ $${\widehat{Y}}_{ABF,h},$$ and $${\widehat{Y}}_{OF,h}$$

These forecasts are aggregated to get the *h*-step-ahead forecasts for the higher level (level 1). Level 1 h-step-ahead forecasts (${\widetilde{Y}}_{AM,h}, {\text{and}} {\widetilde{Y}}_{BF,h}$) are given by

4 $${\widetilde{Y}}_{M,h}={\widehat{Y}}_{AM,h}+{\widehat{Y}}_{BM,h}+ {\widehat{Y}}_{ABM,h}+{\widehat{Y}}_{OM,h}$$

5 $${\widetilde{Y}}_{F,h}={\widehat{Y}}_{AF,h}+{\widehat{Y}}_{BF,h}+ {\widehat{Y}}_{ABF,h}+{\widehat{Y}}_{OF,h}$$

The summing matrix (${\varvec{S}})$ will combine the *h*-step-ahead forecasts up the hierarchical structure. For the bottom-up approach, the forecasts are combined using the formula:

6 $${\widetilde{Y}}_{h}=S{\widehat{Y}}_{K,h},$$

where $$k=0, 1, 2.$$

The advantage of the bottom-up approach is that no information is lost since forecasts are generated at the lowest or base level of the hierarchy. The major setbacks of the method are that, it performs poorly on highly aggregated data and it does not take into account the correlations between the series. Also, too much data points in the base level of the hierarchy requires more runtime to generate forecasts. The bottom-up method is not effective in the case of complex and multi-layered hierarchies [24]. The time series at the lowest levels often have little structure and are therefore difficult to forecast and this can result in forecasting errors which can be aggregated over numerous upper hierarchies.

## The top-down method

This method forecast the highest level of the hierarchy first and then split up the forecast to generate estimates for the lower levels through the use of some proportions or factors. These proportions include average historical proportions, proportions of the historical averages and forecast proportions [16, 25]. Historical data is used in the calculation of the proportions and the approach has the ability to yield reliable forecasts for the aggregate levels [26]. The average historical proportions formula is:

$${p}_{i}= \frac{1}{N}\sum_{t=1}^{N}\frac{{Y}_{i,t}}{{Y}_{t}}$$

where $$i=\mathrm{1,2}, \dots , {m}_{k}.$$ According to [26], every proportion reveals the average of the historical proportions of the bottom level series over time relative to the aggregated series ($${Y}_{t})$$ for $$t=1, 2, 3, . . . , N \left(N=144\right).$$ Using one of the nodes in Fig. 1 and the bottom level series $${Y}_{OF,t}$$ as an example, we can have;

$${p}_{OF}=\left(\frac{{\widehat{y}}_{OF,t}}{{\widehat{S}}_{F,t}}\right)\left(\frac{{\widehat{y}}_{F,t}}{{\widehat{S}}_{Total,t}}\right)$$

where $${\widehat{S}}_{Total,t}={\widehat{Y}}_{M,t}+ {\widehat{Y}}_{F,t}$$ and $${\widehat{S}}_{F,t}={\widehat{y}}_{AF,t}+{\widehat{y}}_{BF,t}+{\widehat{y}}_{ABF,t}+{\widehat{y}}_{OF,t}$$

Advantages of the method is that it provides reliable forecasts for higher levels in the hierarchy and is useful when the lower-level series are noisy and difficult to forecast. The major setback of the method is that there is general loss of information resulting in less accurate forecast being generated at base or lower levels of the hierarchy [27].

## Optimal combination method

Handyman RJ et al. [14] proposed an optimal combination approach for forecasting that utilises all the available information and combinations in a hierarchy. This approach involves making independent forecasts at all levels, which are then reconciled using a linear regression model. The resulting forecasts are coherent and based on weights obtained by solving a system of equations that respect the relationships between the different levels of the hierarchy. This method can estimate the unknown future expectation values of the lowest level of the dataset, K. Given a vector of the unknown means (${{\varvec{\beta}}}_{n}(h))$, thus,

$${{\varvec{\beta}}}_{n}(h)=E[{{\varvec{Y}}}_{k,n+h}|{{\varvec{Y}}}_{1}, {{\varvec{Y}}}_{2},\dots ,{{\varvec{Y}}}_{n}]$$

Since ${{\varvec{Y}}}_{t}$ represents the vector of all observations at time *t* while and ${{\varvec{Y}}}_{k,n+h}$ represents the vector of observations in the bottom level *K*. The base forecasts (${\widehat{{\varvec{Y}}}}_{n}\left(h\right))$ are presented in a regression format to give:

$${\widehat{{\varvec{Y}}}}_{n}\left(h\right)= {\varvec{S}}{{\varvec{\beta}}}_{n}(h)+{{\varvec{\varepsilon}}}_{h}$$

where ${{\varvec{\varepsilon}}}_{h}$ denotes a white noise process with covariance matrix $\sum h$ which is difficult to find in large hierarchies [26]. However, [14] proposed estimating the white noise process by the forecast error in the bottom level, thus,${{\varvec{\varepsilon}}}_{h}\approx {\varvec{S}}{{\varvec{\varepsilon}}}_{k,h}$. With this hypothesis, errors satisfy the same aggregation constraint as the dataset, resulting in

$$\sum h={\varvec{S}}\boldsymbol{ }{\text{Var}}({{\varvec{\varepsilon}}}_{k,h}){{\varvec{S}}}{\prime}$$

The optimal combination approach has a key advantage in that it is capable of producing highly accurate forecasts in comparison to both top-down and bottom-up methods. Additionally, it allows for unbiased forecasts to be generated at all levels while minimising the loss of information. This approach also enables the utilisation of diverse independent forecasting methods, such as ARIMA and ETS, at each level to generate the most accurate forecasts possible. However, one significant drawback of the optimal combination approach is that it can become very complex and computationally intensive when dealing with numerous time series.

## Forecasting individual series

The ETS and ARIMA are the common methods used. The general ARIMA model can be expressed as

$${Y}_{t}-{\Phi }_{1}{Y}_{t-1}-{\Phi }_{2}{Y}_{t-2}-\dots -{\Phi }_{p}{Y}_{t-p}={a}_{t }+{\Theta }_{1}{a}_{t-1 }+{\Theta }_{2}{a}_{t-2 }+\dots +{\Theta }_{q}{a}_{t-q}$$

where $\Phi {\prime}s$ and $\mathrm{\Theta {\prime}}{\text{s}}$ are model parameters.

${Y}_{t}$ – is the stationary series,

${\Phi }_{p}$ – is the coefficient of the *p* ^*th*^ AR term, where p is the order of the AR term,

${\Theta }_{q}$ – is the coefficient of the *q* ^*th*^ MA term, where q is the order of the MA term,

${a}_{t}$—is the error term.

The general forms of the Holt-Winters with permanent constant, linear trend and multiplicative seasonal variations are:

$${\widetilde Y}_t=\alpha\left(\frac{Y_t}{S_{t-s}}\right)+\left(1-\alpha\right)({\widetilde Y}_{t-1}+{\widetilde B}_{t-1}),$$

$${\widetilde B}_t=\beta\left({\widetilde Y}_t-{\widetilde Y}_{t-1}\right)+(1-\beta){\widetilde B}_{t-1,}$$

$$S_t=\gamma\left(\frac{Y_t}{{\widetilde Y}_t}\right)+\left(1-\gamma\right)S_{t-s,}$$

where the smoothing parameters ($\gamma ,$ $\alpha$ and $\beta$) take values between 0 and 1. The smoothed series and seasonality period is denoted ${\widetilde{Y}}_{t}$ and $s$ respectively. Both the ETS and the ARIMA default algorithms are incorporated in the R forecast package HTS. The mean absolute percentage error (MAPE) was used to assess forecasting performance of the models. The MAPE formula is:

$$MAPE=\frac1m\sum\frac{\left[y_t-{\widehat y}_t\right]}{y_t},$$

where ${y}_{t}$ are the actual blood donation values observed, ${\widehat{y}}_{t}$ are predicted blood donation values by the model and $m$ is the prediction period.

### Model validation

The disruptions caused by the Covid-19 pandemic altered blood donation patterns, complicating the forecasting of future blood donations for this specific period of the pandemic. The model validation involved utilising actual blood donation data from January 2019 to December 2020. The model's performance was evaluated through the Mean Absolute Percentage Error (MAPE) suggesting alignment with previous years' donations during the pre-pandemic period, however there MAPE confirmed notable discrepancies between forecasts and observed values during the period of the Covid-19 pandemic.

## Results

### Data and descriptive statistics

Table 1 gives information on the structure of the hierarchy as depicted in Fig. 1.

**Table 1:** Hierarchy of blood donations by gender and blood group types

Level	Type of series	Number of series
Level 0	Total blood donations	1
Level 1	Blood by gender	2 (Male and Female)
Level 2	Blood type	4 (A, B, AB, O)

The descriptive statistics of the data are shown in Table 2.

**Table 2:** Descriptive statistics

	**Variables**	**N**	**Mean**	**Sd**	**Min**	**Max**	**Skew**	**Kurtosis**
A_M_	1	144	725.02	239.74	169	1389	0.21	-0.45
B_M_	2	144	550.95	182.16	128	1055	0.21	-0.45
AB_M_	3	144	115.99	38.38	27	222	0.21	-0.45
O_M_	4	144	1507.85	498.63	351	2888	0.21	-0.45
A_F_	5	144	591.47	192.83	144	1091	0.22	-0.49
B_F_	6	144	449.45	146.57	109	829	0.22	-0.49
AB_F_	7	144	94.69	30.87	23	175	0.22	-0.48
O_F_	8	144	1230.03	401.10	299	2269	0.22	-0.49

*A*_*M*_ Male donor of blood group type A, *B*_*M*_ Male donor of blood group type B, *AB*_*M*_ Male donor of blood group type AB, *O*_*M*_ Male donor of blood group type O, *A*_*F*_ Female donor of blood group type A, *B*_*F*_ Female donor of blood group type B, *AB*_*F*_ Female donor of blood group type AB, *O*_*F*_ Female donor of blood group type O

Monthly mean blood donations for blood type A were 725.02 and 591.47 for males and females, respectively. Blood group O had the highest monthly mean as expected, 1507.85 and 1230.03 for male and female donors respectively. Blood group AB had the least mean donations 115.09 and 94.69 for male and female donors respectively. The negative kurtosis (platykurtic) shows that more donation data are located near the mean and less values are located on the tails thus no cases of extreme values or outliers.

**Fig. 2:**
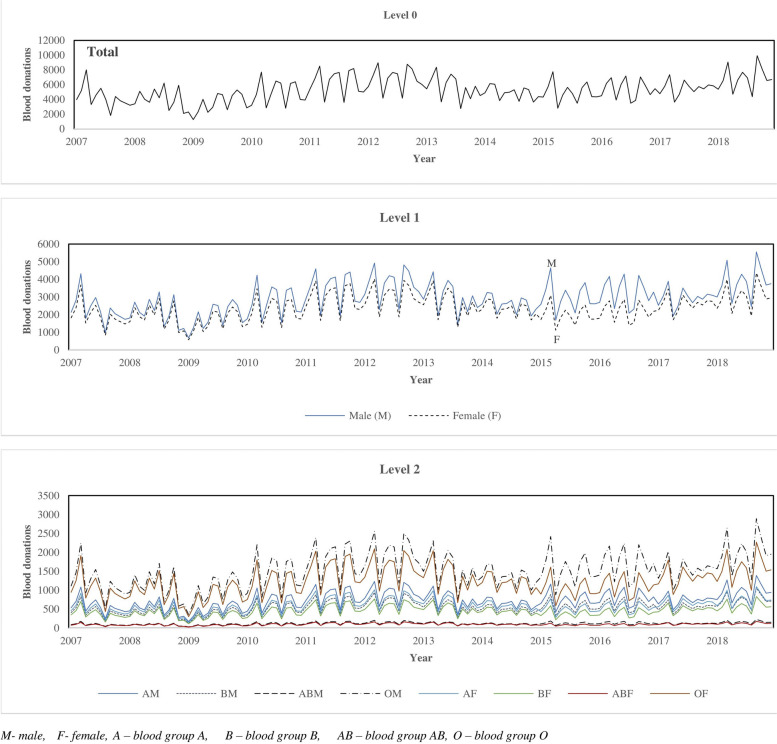
Time series plots based on donor gender and blood group type from 2007 – 2018

The characteristics of the disaggregated blood donations are depicted in Fig. 2.

In Fig. 2, the total blood donations at level 0 exhibit some seasonality. There are no significant variations in the blood donation patterns even though there were some periods of declines in blood donations. At level 1, the male donations (M) surpassed their female counterparts (F). It is evident from Fig. 2 that blood group O donations (OM and OF) have the highest volumes, followed by blood group A and blood group AB being the least. At level 2, male blood group O had a maximum of 2888 units, female blood group O had a maximum of 2269 units and female blood group AB had the least maximum of 175 units. Such insights help blood centre authorities to plan for blood donor education and recruitment scheduling, fixed and mobile drives, blood collection and also meeting clinical blood transfusion needs.

## Forecasting accuracy evaluation

The MAPE accuracy measure was used to assess the forecasting performance of the models. An out-of-sample forecasting accuracy measure is done. Table 3 presents accuracy done for both the ETS and ARIMA as forecasting methods.

**Table 3:** Forecast error measures (MAPE)

	**ETS Forecasting method**					**ARIMA Forecasting method**				
	**BU**	**TDFP**	**TDHA**	**TDHP**	**OC**	**BU**	**TDFP**	**TDHA**	**TDHP**	**OC**
**Total**	12.58	12.43	12.43	12.43	12.57	11.27	11.42	11.42	11.42	11.42
**M (Male)**	12.68	11.32	11.4	12.52	12.66	12.18	12.45	10.68	10.62	10.62
**F (Female)**	12.45	14.03	13.9	12.32	12.45	11.97	10.11	12.37	12.44	12.44
**A**_**M**_	12.81	11.32	11.39	12.65	12.8	12.17	12.44	10.67	10.61	10.61
**B**_**M**_	12.76	11.32	11.39	12.6	12.75	12.21	12.48	10.69	10.63	10.63
**AB**_**M**_	12.62	11.33	11.41	12.46	12.62	12.15	12.43	10.69	10.63	10.63
**O**_**M**_	12.59	11.32	11.39	12.43	12.57	12.17	12.45	10.68	10.62	10.62
**A**_**F**_	12.44	14.04	13.91	12.31	12.44	11.95	10.1	12.37	12.44	12.44
**B**_**F**_	12.42	14.02	13.89	12.29	12.42	11.96	10.1	12.36	12.43	12.43
**AB**_**F**_	12.56	13.99	13.86	12.43	12.56	12.04	10.24	12.42	12.49	12.49
**O**_**F**_	12.46	14.03	13.9	12.33	12.46	11.98	10.12	12.37	12.44	12.44
**Average**	12.58	12.65	12.62	12.43	12.57	12	**11.3**	11.52	11.52	11.52

*BU *Bottom-up approach, *TDHP *Top-down approach with average historical proportions

*TDHA *Top-down approach with proportion of historical averages

*TDFP *Top-down approach with forecast proportions and OC-optimal combination approach

**Fig. 3:**
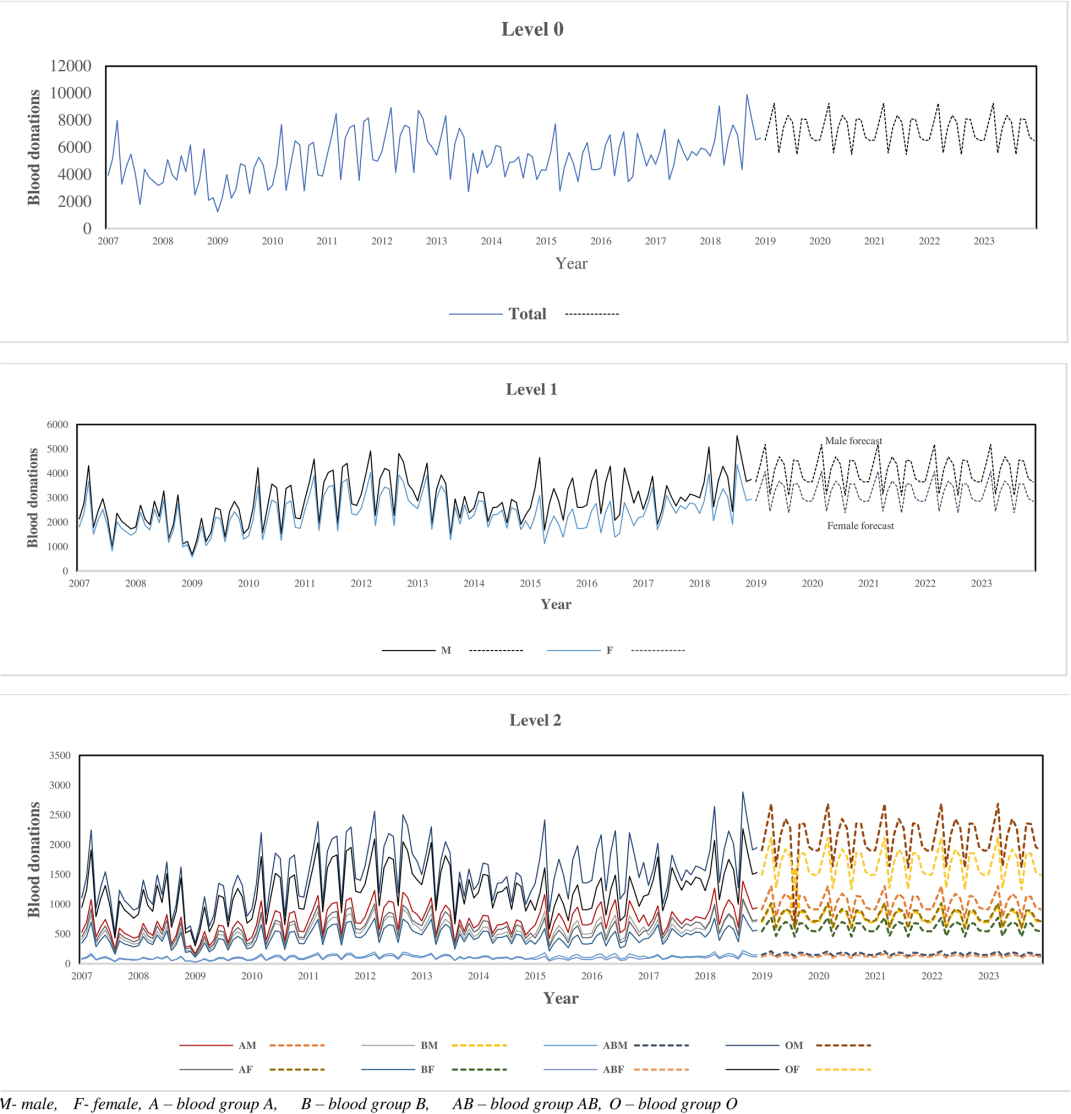
Blood donation forecasts from 2019 – 2023 using TDFP under ARIMA

The average accuracy measures from each model are under the row named “Average”. It is shown in Table 3 that the TDFP approach produces small MAPE values under the ARIMA forecasting method forecasting method. The TDFP under ARIMA with MAPE error of 11.30 is the best and is used to forecast future blood quantities. Table 4 and Fig. 3 show out-of-sample forecasted future values for 60 months and their graphical display.

**Table 4:** Out-of-sample future blood forecasts

Month	Total	M	F	AM	BM	ABM	OM	AF	BF	ABF	OF
Jan-19	6540	3664	2875	913	698	148	1906	721	544	116	1496
Feb-19	7876	4405	3471	1101	837	176	2292	863	659	140	1811
Mar-19	9268	5187	4081	1304	986	209	2688	1020	775	165	2121
Apr-19	5610	3157	2453	792	600	128	1638	614	466	98	1274
May-19	7464	4187	3277	1052	798	168	2169	818	624	132	1705
Jun-19	8377	4687	3690	1175	890	187	2435	922	701	148	1920
Jul-19	7865	4387	3478	1098	832	175	2282	873	664	139	1802
Aug-19	5496	3101	2395	774	1614	123	590	597	456	96	1245
Sep-19	8117	4545	3572	1136	869	183	2357	894	678	144	1857
Oct-19	8075	4510	3565	1127	854	181	2349	893	677	143	1853
Nov-19	6748	3782	2966	947	722	150	1964	743	562	119	1543
Dec-19	6507	3649	2858	911	692	147	1900	712	544	115	1488
Jan-20	6540	3664	2876	913	698	148	1906	721	544	115	1496
Feb-20	7876	4405	3471	1101	836	176	2292	863	659	140	1811
Mar-20	9267	5187	4081	1304	986	209	2688	1020	775	165	2121
Apr-20	5610	3157	2453	792	600	128	1638	614	466	98	1275
May-20	7464	4187	3277	1052	798	167	2169	818	624	132	1704
Jun-20	8377	4687	3690	1175	890	187	2435	922	701	148	1920
Jul-20	7865	4387	3478	1098	832	175	2282	873	664	139	1802
Aug-20	5496	3101	2395	774	590	123	1614	598	456	96	1246
Sep-20	8117	4545	3572	1136	869	183	2357	894	678	144	1857
Oct-20	8075	4510	3565	1127	854	180	2349	893	677	143	1853
Nov-20	6748	3782	2966	946	722	150	1964	743	562	119	1543
Dec-20	6507	3649	2858	911	692	147	1900	712	544	115	1488
Jan-21	6540	3664	2876	913	698	148	1906	721	544	116	1496
Feb-21	7876	4405	3471	1101	836	176	2292	863	659	140	1811
Mar-21	9268	5187	4081	1304	986	209	2688	1020	775	165	2121
Apr-21	5610	3157	2453	792	600	128	1638	614	466	98	1275
May-21	7464	4187	3277	1052	798	167	2169	818	624	132	1705
Jun-21	8377	4687	3690	1175	890	187	2435	922	701	148	1920
Jul-21	7865	4387	3478	1098	832	175	2282	873	664	139	1802
Aug-21	5496	3101	2395	774	590	123	1614	597	456	96	1246
Sep-21	8117	4545	3572	1136	869	183	2357	894	678	144	1857
Oct-21	8075	4510	3565	1127	854	180	2349	893	677	143	1853
Nov-21	6748	3782	2966	946	722	150	1964	743	562	119	1543
Dec-21	6507	3649	2858	911	692	147	1900	712	544	115	1488
Jan-22	6540	3664	2876	913	698	148	1906	721	544	116	1496
Feb-22	7876	4405	3471	1101	836	176	2292	863	659	140	1811
Mar-22	9268	5187	4081	1304	986	209	2688	1020	775	165	2121
Apr-22	5610	3157	2453	792	600	128	1638	614	466	98	1275
May-22	7464	4187	3277	1052	798	167	2169	818	624	131	1705
Jun-22	8377	4686	3691	1175	890	187	2435	922	701	148	1920
Jul-22	7865	4387	3478	1098	832	175	2282	873	664	139	1802
Aug-22	5496	3101	2395	774	590	123	1614	597	456	96	1246
Sep-22	8117	4545	3572	1136	869	183	2357	894	678	144	1857
Oct-22	8075	4510	3565	1127	854	180	2349	893	677	143	1853
Nov-22	6749	3782	2966	946	722	150	1964	743	562	119	1543
Dec-22	6507	3649	2858	911	692	147	1900	712	544	115	1488
Jan-23	6540	3664	2876	913	698	148	1906	721	544	116	1496
Feb-23	7876	4405	3471	1101	837	176	2292	863	659	140	1811
Mar-23	9268	5187	4081	1304	986	209	2688	1020	775	165	2121
Apr-23	5610	3157	2454	792	600	128	1638	614	466	98	1275
May-23	7464	4187	3277	1052	798	168	2169	818	624	131	1705
Jun-23	8377	4687	3691	1175	890	187	2435	922	702	148	1920
Jul-23	7865	4387	3478	1098	832	175	2282	873	664	140	1802
Aug-23	5496	3101	2395	774	590	123	1614	598	456	96	1246
Sep-23	8117	4545	3572	1136	870	183	2357	894	678	144	1857
Oct-23	8075	4510	3565	1127	854	181	2349	893	677	143	1853
Nov-23	6749	3783	2966	946	722	150	1964	743	562	119	1543
Dec-23	6507	3649	2858	911	692	147	1900	712	544	115	1488

*M *male*, F *Female,  *A *Blood group,  *AB *Blood group*, AB* blood group AB*, O *Blood group O

From Fig. 3, future blood donations forecasts are indicated by the dashed/dotted line(s) while the historical data are represented by solid line(s). At level 1, future projections show that, male donations are higher than female donations. Similarly, at level 2, the projected donations for blood group O for males (OM) are higher than for their female (OF) counterparts. It is evident from all the three-levels in Fig. 3 that there could be a steady to slight decline in future blood donations for all the donor categories based on the projections. This can be attributed to a real problem of a continuous decline in numbers of regular voluntary blood donors in most blood centres. Low blood donations for blood group AB are projected to continue in the short to long term periods. This calls for the development of sound policies and interventions in blood donor and blood management (Table 5).

**Table 5:** Actual and forecast blood donations for 2019 and 2020

	**Blood Donations**			
**Month**	**Actual 2019**	**Forecast 2019**	**Actual 2020**	**Forecast 2020**
January	5724	6540	5600	6540
February	8299	7876	7900	7876
March	10,812	9268	8850	9267
April	7519	5610	2350	5610
May	9067	7464	2000	7464
June	10,439	8377	3500	8377
July	9493	7865	2550	7865
August	6418	5496	3700	5496
September	8474	8117	3850	8117
October	6586	8075	5600	8075
November	5914	6748	5950	6748
December	7131	6507	5900	6507
	MAPE = 14.80		MAPE = 84.06	

The blood donation predictions for 2019 had a MAPE value of 14.80, suggesting alignment with previous years' donations. However, starting in April 2020, the Covid-19 pandemic disrupted blood collection, leading to a significant decrease in blood donation and hence a decrease in model accuracy, and this is then reflected in a high MAPE value of 84.06.

## Discussion

The aim of this study was to explore blood donation forecasting technique that could generate accurate and coherent predictions. The blood donation data in Zimbabwe recorded by the NBSZ was categorised according to donor specific characteristics. These categories gave rise to hierarchical time series forecasting. The forecasts from the approach suggest the need for effective donor education and recruitment drives targeting blood group O donors since they are universal blood donors and blood type O is always on high demand. These methods are strongly recommended as they give feasible solutions. They capture blood donor data dynamics, produce precise and sensible forecasts.

Previous studies have attributed patterns in blood supply to socio-demographic characteristics [28–30]. These donor specific characteristics give rise to clusters warranting the application of hierarchical forecasting. Therefore, there is need to make blood donation projections based on blood donor socio-demographic characteristics.

A study by [31] projected future blood donors in Birjand City, Iran using decision trees. Their models yielded poor performance based on the measures of accuracy. They concluded that the trees had numerous disaggregation of the data leading to data overfitting. Results from the current study indicated that the data disaggregation helped in generating accurate and coherent forecasts.

A time series analysis by aggregating blood donation frequency by month was conducted by [32]. The study results showed a stable blood supply for most months except in June and September periods which coincide with religious festivals in Saudi Arabia. The current study results showed seasonality in the donation patterns. The seasonality is linked to public holiday months and school holidays in Zimbabwe during the months of April, August and December each year.

Forecasting blood donation based on blood group prevalence is vital in managing blood supply at a blood bank [33]. Keeping track of dynamic changes in the donation prevalence of different blood groups is important since the distribution of the blood groups varies with time [34, 35]. Also, [36] concluded that blood donors with blood group O had higher frequency of blood donations and a lesser risk of lapsing, leading to the need for high blood donation volumes compared to other blood groups.

The current study shows that blood donations from blood group O donors have the highest volumes of donations compared to the other blood groups. This can be associated with the fact that the proportion of blood group O is highest in the donor population in Zimbabwe (52%). At the same time, blood group O donors are referred to as universal donors because blood type O can be transfused to blood A and B patients in emergencies where there was no time for matching blood types. However, it is current best practice to transfuse group-specific blood. Such insights help blood centre authorities to plan for blood donor education and recruitment, schedule blood drives and blood collections and also meeting clinical blood transfusion needs.

The results from the blood donation projections by gender concurs with other researchers where male donations are consistently higher than their female donations [37–39]. The current study also shows similar trends where male blood donors had higher mean blood donations compared to the female donors. Males have a higher frequency of donations as they are allowed, through regulation, to donate blood after every 12 weeks compared to 16 weeks interval for the female donors. Other researchers have attributed the lower donation volumes of female donors to high donor lapsing compared to male donors [36, 40].

Women generally donate blood less than men due to deferrals as a result of iron depletion through menstrual blood loss.

The blood donation projections from the study have some clinical implications. Some previous studies have shown that the survival rate of patients transfused with blood from male donors was higher compared to female donors [41]. Therefore, the higher proportion of male donors in the pool is vital in clinical blood transfusion. Also, Zimbabwe often experiences shortages in blood group type O. Therefore, the higher proportion of blood group O donors in the projections will help blood authorities in rationalising blood donor education and recruitment to minimise blood shortfalls.

Blood collections trend took a down turn from April 2020 as the government of Zimbabwe introduced Covid-19 lockdown restrictions to reduce the spread of the pandemic. These measures rendered most blood collection sites inaccessible as movement of people was restricted. The NBSZ had to rely on community based and walk in blood donors and this resulted in a 40% decrease in units of blood collected compared to 2019. The same negative impact of the Covid-19 pandemic can be observed from 2021 up to June 2022. This means that alternative models could be developed in future studies to analyse the impact of pandemics in forecasting blood donations. A time series with intervention model would be an ideal alternative candidate. The model focuses on the shock or pulse that results after say, a pandemic.

## Conclusion

The discrepancy between blood supply and demand and the perishability of blood and blood components can be alleviated somewhat through accurate forecasts of the blood supply at any blood bank. Such accurate and coherent forecasts help in safeguarding the risks of understocking and overstocking the scarce and perishable resource, blood. Thus, accurate statistical forecasting methods play a significant role in future blood donation projections. The top-down, bottom-up and optimal combination approaches were adopted in the study with each approach having its own merits and demerits. The EST and ARIMA methods were used to generate the forecasts. The TDFP under ARIMA with the smallest MAPE was considered to be the best and was then used to forecast future blood donations.

Future blood forecasts indicated a slight decrease in total blood donations. This suggests the need for blood centre authorities to develop sound blood donor management interventions. Such interventions include an integrated strategy of the entire blood safety value chain, including donor education, targeted recruitment and retention, scheduled fixed and mobile blood donation drives, safe blood collection and donor care and adequate resource allocation.

Study results showed that blood donations from blood group O donors have the highest volumes of donations compared to the other blood groups. Also, blood donations by the male gender are higher than donations by their female counterparts. These trends are attributed to the higher proportions of donors in these categories.

This study will contribute to the board of knowledge on the adoption of coherent and accurate hierarchical forecasting methods in ensuring an adequate and safe blood supply chain in a low resource setting like Zimbabwe.

This study has potential limits. The lack of prior research studies on the topic limited the scope of the current study. The impact of the Covid-19 pandemic distorted the blood donation patterns such that the developed model did not capture the significant drop in blood donations during the pandemic period. Other shocks such as, a surge in global pandemics and other disasters, will inevitably affect the blood donation system. This means that future blood supplies remain under threat. Thus forecasting future blood collections with a high degree of accuracy requires robust mathematical models which factor in the impact of various shocks to the system on short notice. Door to door blood donation drives are not out of the question in such instances .

## Data Availability

The data that support the findings of this study are available from the corresponding author and the National Blood Service Zimbabwe upon reasonable request.

## Abbreviations

NBSZ: National Blood Service Zimbabwe
ETS: Error-Trend-Seasonality
ARIMA: Autoregressive Integrated Moving Average
MAPE: Mean Absolute Percentage Error
TDFP: Top-Down Forecasting Proportions
FS: Fuzzy Systems
ANN: Artificial Neural Networks
SVM: Support Vector Machine
HTS: Hierarchical Time Series
MSE: Mean Square Error
MAE: Mean Absolute Error
